# Implications of *Peptidyl Arginine Deiminase 4* gene transcription and polymorphisms in susceptibility to rheumatoid arthritis in an Iranian population

**DOI:** 10.1186/s12920-023-01532-9

**Published:** 2023-05-16

**Authors:** Zahra Bagheri-Hosseinabadi, Mohammad Reza Mirzaei, Ozrasadat Esmaeili, Fatemeh Asadi, Hassan Ahmadinia, Banafshe Shamsoddini, Mitra Abbasifard

**Affiliations:** 1 Molecular Medicine Research Center, Research Institute of Basic Medical Sciences, Rafsanjan University of Medical Sciences, Rafsanjan, Iran; 2 Department of Clinical Biochemistry, School of Medicine, Rafsanjan University of Medical Sciences, Rafsanjan, Iran; 3 Occupational Environmental Research Center, Medical School, Rafsanjan University of Medical Sciences, Rafsanjan, Iran; 4 Student Research Committee, Rafsanjan University of Medical Sciences, Rafsanjan, Iran; 5 Department of Internal Medicine, Ali-Ibn Abi-Talib Hospital, School of Medicine, Rafsanjan University of Medical Sciences, Rafsanjan, Iran

**Keywords:** Rheumatoid arthritis, Peptidyl arginine deiminase 4, Single nucleotide polymorphism, Genetic association

## Abstract

**Background:**

Peptidyl arginine deiminase 4 (PADI4) has been implicated in Rheumatoid arthritis (RA) pathogenesis. Here we aimed to evaluate the association of *PADI4* gene rs11203367 and rs1748033 single nucleotide polymorphisms (SNPs) with RA proneness.

**Methods:**

The mRNA expression of PADI4 was determined in the whole blood samples. The genotyping of *PADI4* polymorphisms was conducted using allelic discrimination TaqMan genotyping Real-time PCR.

**Results:**

The alleles and genotypes of rs11203367 polymorphism were not associated with susceptibility to RA risk. The T allele (OR = 1.58, 95%CI: 1.21–2.04, *P* = 0.0005), TT genotype (OR = 2.79, 95%CI: 1.53–5.06, *P* = 0.0007), TC genotype (OR = 1.52, 95%CI: 1.04–2.23, *P* = 0.0291), dominant (OR = 1.72, 95%CI: 1.19–2.47, *P* = 0.0034) and recessive (OR = 2.19, 95%CI: 1.25–3.82, *P* = 0.0057) models of rs1748033 SNP were associated with higher risk of RA. There was a significant upregulation of PADI4 mRNA in the RA patients compared to controls. mRNA expression of PADI4 had significantly positive correlation with anti-CCP level (*r* = 0.37, *P* = 0.041), RF level (*r* = 0.39, *P* = 0.037), and CRP level (*r* = 0.39, *P* = 0.024).

**Conclusion:**

*PADI4* gene rs1748033 SNP was associated with increased RA risk. This polymorphism might affect the RA pathogenesis regardless of impressing the levels of PADI-4 in serum.

## Introduction

Rheumatoid arthritis (RA) is defined as a systemic autoimmune and autoinflammatory disorder that causes a chronic inflammation in the synovium culminates in a destruction of joints. RA affects approximately over 1% of the general population worldwide with higher incidence risk in women compared to men. Based on a bulk of evidence, the interaction between environmental factors and genetics are involved in pathomechanism of RA [1–4]. It has been shown that the genetic abnormalities account for approximately 60% of susceptibility to RA with at least 30% attributed to the genes in the Human leukocyte antigen (HLA) class II locus [5–8].

Among the non-HLA gene, studies have shown the association of single nucleotide polymorphisms (SNPs) in the *peptidyl arginine deiminase 4* (*PADI4*) gene with RA predisposition [9]. PADI4 is an enzyme that facilitates the conversion of arginine residues in the proteins to citrulline over the post-translational modifications of proteins [10, 11]. Reports show the increased levels of citrullinated proteins in the synovial fluid samples obtained from RA subjects. In addition, antibodies against citrullinated proteins (anti-CCP) are prevalent in the blood of RA patients and is highly specific for diagnosis of RA. Research indicate that anti-CCP antibodies have predictive potential for RA development and have been related with the disease severity of patients with RA [12].

Genetic association of SNPs in *PADI4* gene with RA risk have been indicated in several populations. Earlier reports by Suzuki and colleagues in 2003 demonstrated first evidence of the association between *PADI4* SNPs and predisposition to RA in the Japanese patients [13]. Following this, several investigations in various populations (such as North American, East Asian, and German populations) have been conducted that validated the association between *PADI4* gene SNPs and RA risk. That notwithstanding, inconsistent reports were shown in patients from European populations [14–16]. Studies performed on UK, Swedish, and Spanish patients did not show significant associations between *PADI4* gene polymorphisms and RA risk [17–19]. The 2006 and 2007 meta-analysis studies indicated that *PADI4* gene SNPs were associated with RA proneness in the European as well as Asian populations [20, 21]. Furthermore, the 2013 meta-analysis on the studies performed in the Egyptian and Chinese populations revealed that the *PADI4* gene SNPs were associated with RA risk [22]. A study in Iranian population did not show association of *PADI4* gene rs11203367 and rs874881 polymorphisms with RA risk [23]. However, a study on the RA population from Southeast Iran indicated that *PADI4* rs1748033 T allele as well as the dominant and codominant inheritance models were associated with increased RA risk [24].

Observations about the association of *PADI4* gene rs11203367 and rs1748033 SNPs and RA are controversial. Moreover, the genetic stratification of different ethnic groups differs, which are effective regarding the personalized medicine scheme, prioritizing genotyping of the subjects in different geo-epidemiological regions (Rafsanjan city in our case here) order to attaining most of the available therapeutics. As a consequence, here we aimed to assess the association of *PADI4* gene rs11203367 and rs1748033 SNPs with susceptibility to RA. Moreover, the effect of these SNPs on the mRNA expression of PADI4 were determined.

## Subjects and methods

### Study participants

In this case-control investigation, 250 RA patients were recruited from the subjects referred to the Rheumatology clinic of the Ali-Ebne-Abitaleb Hospital, Rafsanjan, Kerman, Iran. The diagnosis of patients were conducted based on the American College of Rheumatology (ACR) / European Alliance of Associations for Rheumatology (EULAR) criteria for RA diagnosis and classification [25]. Clinical presentations of the patients, such as number of tender swollen joints, morning stiffness, and extra-articular signs were collected for all patients. In addition, disease activity of the RA patients was measured by determining Disease Activity Score 28 (DAS28) for each subject. Patients were excluded from the study if they had other chronic inflammatory diseases either in themselves or close family members, pregnancy, allergy, etc. We also included 250 age- and gender-matched healthy individuals as the control group who did not have autoimmune and autoinflammatory disorders as well as familial history of autoimmunity, immunodeficiency, and malignancy. This work was performed based on the Declaration of Helsinki for studying the human subjects. The study was approved by the Ethics Committee from Rafsanjan University of Medical Sciences and informed approval was obtained from all patients before initiation of study. Ultimately, 5 ml of peripheral blood samples was obtained in the EDTA-coated vials to perform molecular analysis.

### RNA isolation and cDNA synthesis and real-time PCR

Serum of all 250 patients and 250 healthy controls were isolated using centrifugation. RNA extraction from plasma was conducted using Trizol total RNA extraction kit (GeneAll, Korea) according to manufactures’ instructions. Determination of the relative absorbance ratio at A260/A280 and A260/A230 by a spectrophotometer (Nano Drop 2000, Thermo Scientific, USA) was exerted to assess the extracted RNA concentration and purity. Then, template RNA was reverse-transcribed by PrimeScript 1st strand cDNA Synthesis Kit (TAKARA, Japan) following the company’s guidelines using Thermal Cycler instrument (Eppendorf, Germany). Real-time mRNA expression of PADI4 was conducted using ABI StepOnePlus real-time PCR System (Applied Biosystems, Foster City, CA, USA) and CYBR Green MasterMix using specific primers (Table 1). The Real-time analyses were conducted in triplicate order. The transcript level of Glyceraldehyde 3-phosphate dehydrogenase (GAPDH) was measured as housekeeping gene to normalize the expression levels of target genes. The comparative threshold cycle method (2^−∆∆ct^) was exerted to measure the relative amounts of target genes in each sample.

**Table 1:** Primers used to determine the mRNA expression of PADI4.

Gene		Gene Sequence (5′→3′)
PADI4	Forward	5′-GCACAACATGGACTTCTACGTGG-3′
	Reverse	5′-CACGCTGTCTTGGAACACCACA-3′
GAPDH	Forward	5′-TGCCACTCAGAAGACTGTGG-3′
	Reverse	5′- GGATGCAGGGATGATGTTCT-3′

PADI4; Peptidyl Arginine Deiminase 4, GAPDH; Glyceraldehyde 3-phosphate dehydrogenase

### SNP genotyping

In order to perform genotyping, DNA was extracted from the whole blood samples of 250 patients and 250 healthy controls using the phenol-chloroform procedure [26]. The quality and concentration of the extracted DNA was determined using a Nano Drop spectrophotometer (Nano Drop 2000, Thermo Scientific, USA). All samples were genotyped for *PADI4* gene rs11203367 and rs1748033 SNPs using Real-time allelic discrimination TaqMan assays (Applied Biosystems, Foster City, USA) by StepOnePlus Real-Time PCR system (Applied Biosystems, Foster City, USA). For performing PCR amplification, each reaction mixture contained 2 µl of extracted DNA (with 20 ng/µl concentration), 5 µl of TaqMan Master Mix (containing Taq DNA polymerase and dNTPs), 0.5 µl TaqMan Genotyping Assay mix (containing pre-designed primers and FAM or VIC labeled probes; Applied Biosystems, Foster City, USA), and distilled water to the ultimate volume of 20 µl. The thermocycling conditions for the PCR amplification were: initial heating at 60 °C for 45 s and then 95 °C for 10 min, and then 40 cycles of amplification by 95 °C for 15 s and 60 °C for 1 min, and finally 60 °C heating for 50 s. Determination of alleles in each sample was conducted via analysis of the allelic discrimination plots through ABI SDS Version 2.2 software.

### Statistical analysis

The generalized linear model (i.e., logistic regression) was used to assess the association of alleles and genotypes of *PADI4* gene rs11203367 and rs1748033 SNPs with RA risk. Additionally, the chi-square test was utilized to measure the association of alleles and genotypes with the clinical manifestations of the RA patients. Odds ratios (ORs) with 95% confidence interval (95% CI) were determined to indicated the effect size. Using the SHEsis online tool [27], the control group was tested for Hardy–Weinberg Equilibrium (HWE). The normality of distribution for the quantitative variables was assessed by Kolmogorov–Smirnov test. The difference of scale variables between groups was tested by independent sample *t*-test or Mann-Whitney *U* test where applicable. Correlation analyses were performed by either Pearson’s or Spearman’s tests. All statistical analyses were performed by the Statistical Program for Social Science (SPSS) v. 23 software. Scale data were shown as the mean ± standard deviation (SD) and the qualitative data were exhibited as numbers or percentages. The level of the statistical significance was set as a *P* value < 0.05.

## Results

### Baseline data and demographics of the study subjects

Demographic and laboratory information of RA patients and healthy controls are shown in Table 2. The study group was comprised of 250 RA patients containing 205 (82%) females and 45 (18%) males as well 250 healthy controls involving 202 (80.8%) females and 48 (19.2%) males. The age of RA cases and healthy controls were 53.15 ± 10.89 and 49.2 ± 15.7 years, respectively. Therefore, patient and control groups were matched for gender and age as well as ethnicity. In each RA and control group, there were 44 (17.6%) and 206 (82.4%) cases, respectively, who were smoking. Regarding laboratory data, anti-CCP level was 58.74 ± 85.19 mg/L, Rheumatoid factor (RF) level was 41.07 ± 33.1 mg/L, Erythrocyte sedimentation rate (ESR) was 21.63 ± 18.8 mm/h, and C-reactive protein (CRP) level was 3.47 ± 2.63 mg/L. The disease activity of RA cases was measured by DAS-28 score, which was 4.83 ± 1.01 for our RA population in this study.

**Table 2:** Baseline data and clinical characteristics of the study subjects

Feature	RA patient (n = 250)	Healthy control (n = 250)	*P* value
**Age**	53.15 ± 10.89	49.2 ± 15.7	> 0.05
**Gender**			
Female	205 (82%)	202 (80.8%)	> 0.05
Male	45 (18%)	48 (19.2%)	
**Smoking**			
Yes	44 (17.6%)	41 (16.4%)	> 0.05
No	206 (82.4%)	209 (83.6%)	
**BMI (Kg/m** ^**2**^ **)**	29.41 ± 4.90	-	-
**Anti-CCP (mg/L)**	58.74 ± 41.19	-	-
**RF level (mg/L)**	41.07 ± 33.1	-	-
**ESR (mm/h)**	21.63 ± 18.8		-
**CRP (mg/L)**	3.47 ± 2.63	-	-
**DAS28**	4.83 ± 1.01	-	-
**Corticosteroid use; n (%)**	185 (74%)	-	-
**DMARD use; n (%)**	138 (55.2%)	-	-
**NSAID use; n (%)**	211 (84.4%)	-	-

RA; Rheumatoid arthritis, BMI; Body-mass index, Anti-CCP; Anti-cyclic Citrullinated Peptide Antibody, RF; Rheumatoid factor, DAS28; Disease Activity Score in 28 Joints, ESR; Erythrocyte sedimentation rate, CRP; C-reactive protein, DMARD; Disease-modifying antirheumatic drugs, NSAID; Non-steroidal anti-inflammatory drugs

### mRNA expression of PADI4

**Fig. 1:**
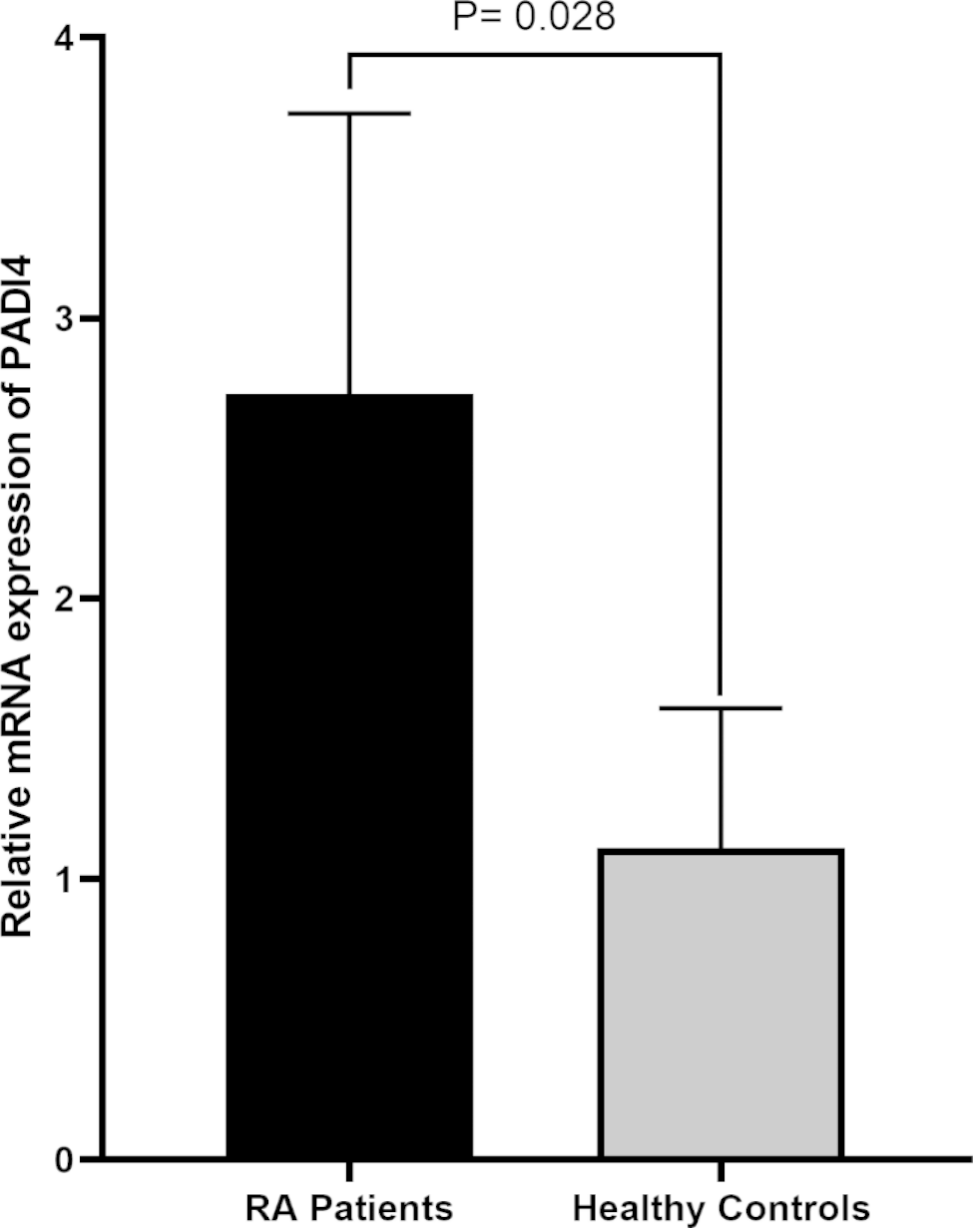
The mRNA expression of PADI4 in RA patients was 4.45-fold higher than the control group. There was a significant upregulation of PADI4 in the patients compared to controls (*P* = 0.028)

It was seen that the mRNA expression of PADI4 in RA patients was 4.45- fold higher than the control group. There was a significant upregulation of PADI4 in the patients compared to controls (*P* = 0.028, Fig. 1).

We also compared the smoker and non-smoker as well as the male and female RA subjects regarding mRNA expression of PADI4, but no statistically significant results were found (data not shown).

### Allele and genotype frequency

Analyses indicated that the distribution of genotype frequencies for rs11203367 (*P* = 0.729) and rs1748033 (*P* = 0.246) polymorphisms did not deviate from HWE (Table 3).

**Table 3:** Allele and genotype frequencies of *PADI4* gene rs11203367 and rs1748033 SNPs in RA patients and healthy controls and corresponding association analyses

dbSNP	Frequency				
	RA patients (N = 250)			Controls (N = 250)	
rs11203367					
T	223 (44.6%)			216 (43.2%)	
C	277 (55.4%)			284 (56.8%)	
TT	51 (20.4%)			48 (19.2%)	
TC	121 (48.4%)			120 (48%)	
CC	78 (31.2%)			82 (32.8%)	
HWE for controls = 0.729					
**Association Test**					
**Model**		**OR**	**95% CI**		***P*** **value**
Allele	T vs. C	1.05	0.82–1.35		0.6541
Codominant	TT vs. CC	1.11	0.67–1.84		0.6652
Codominant	TC vs. CC	1.06	0.71–1.58		0.7751
Dominant	TT + TC vs. CC	1.07	0.73–1.56		0.7015
Recessive	TT vs. TC + CC	1.07	0.69–1.67		0.7364
**rs1748033**					
T	211 (42.4%)			158 (31.6%)	
C	289 (57.8%)			342 (68.4%)	
TT	42 (16.8%)			21 (8.4%)	
TC	127 (50.8%)			116 (46.4%)	
CC	81 (32.4%)			113 (45.2%)	
HWE for controls = 0.246					
**Association Test**					
**Model**		**OR**	**95% CI**		***P*** **value**
Allele	T vs. C	1.58	1.21–2.04		0.0005
Codominant	TT vs. CC	2.79	1.53–5.06		0.0007
Codominant	TC vs. CC	1.52	1.04–2.23		0.0291
Dominant	TT + TC vs. CC	1.72	1.19–2.47		0.0034
Recessive	TT vs. TC + CC	2.19	1.25–3.82		0.0057

SNP; Single nucleotide polymorphism, RA; Rheumatoid arthritis, OR; odds ratio, 95% CI; 95% confidence interval, HWE; Hardy-Weinberg equilibrium

The C allele of rs11203367 SNP was considered as the reference allele (Table 3). It was seen that the T allele of this SNP had higher frequency in RA patients and healthy controls (44.6% vs. 43.2%); however, the difference was not significant (OR = 1.05, 95%CI: 0.82–1.35; *P* = 0.65). The CC genotype of rs11203367 SNP was set as the reference genotype. The TT genotype of rs11203367 SNP was frequent in RA patients than in controls (20.4% vs. 19.2%); however, the difference was not statistically significant (OR = 1.11, 95% CI: 0.67–1.84; *P* = 0.66). The TC genotype had almost similar frequency in both RA and control groups (48.4% vs. 48%), hence there was no statistically significant difference in the prevalence of this genotype between the two groups (OR = 1.06, CI: 0.71–1.58; *P* = 0.77). Both dominant and recessive models were not associated with RA risk.

For rs1748033 SNP, the wild type C allele was considered as the reference allele. It was detected that the T allele of this SNP was more prevalent in the RA group compared to the controls (42.4% vs. 31.6%) and it was associated with a significant increased risk of RA (OR = 1.58, 95%CI: 1.21–2.04, *P* = 0.0005). Additionally, the major CC genotype was regarded as the reference genotype. The TT genotype was seen to be highly prevalent in the RA patients compared to the controls (16.8% vs. 8.4%) and it was associated with an increased RA risk (OR = 2.79, 95%CI: 1.53–5.06, *P* = 0.0007). The heterozygote TC genotype was highly expressed in the RA patients than healthy subjects (50.8% vs. 46.4%) and this genotype had significant association with increased risk of RA (OR = 1.52, 95%CI: 1.04–2.23, *P* = 0.0291). The dominant (TT + TC vs. CC; OR = 1.72, 95%CI: 1.19–2.47, *P* = 0.0034) and recessive (TT vs. TC + CC; OR = 2.19, 95%CI: 1.25–3.82, *P* = 0.0057) models were associated with higher risk of RA (Table 3).

The analyses were also conducted based on smoking and gender status of RA subjects, but no statistically significant results were obtained (data not shown).

### Correlation analysis

Correlation analysis was conducted between mRNA expression of PADI4 and age, body mass index (BMI), anti-CCP, RF, ESR, CRP, and DAS28 in 250 RA cases (Table 4). It was observed that mRNA expression of PADI4 had significantly positive correlation with anti-CCP level (*r* = 0.37, *P* = 0.041). PADI4 mRNA expression was positively correlated with RF level in the RA patients (*r* = 0.39, *P* = 0.037). There was a significantly direct correlation between mRNA expression of PADI4 and CRP level (*r* = 0.39, *P* = 0.024). PADI4 mRNA expression had a statistically significant correlation with DAS28 score in the RA cases (*r* = 0.44, *P* = 0.001).

**Table 4:** Correlation analysis between mRNA expression on PADI4 and clinical manifestations of 250 RA patients

Characteristic	Correlation coefficient	*P* value
Age (years)	0.24	0.847
BMI (kg/m^2^)	0.13	0.675
Anti-CCP (IU/ml)	0.37	**0.041**
RF	0.39	**0.037**
ESR	0.28	0.081
CRP	0.39	**0.024**
DAS28	0.44	**0.001**

BMI; Body mass index, DAS28; Disease Activity Score 28, RF; Rheumatoid factor, Anti-CCP; Anti-Cyclic Citrullinated Peptide Antibody

Correlation analysis was performed in the smoker/non-smoker and male/female subjects to obtain more precise outcome, but the results were not statistically significant (data not shown).

### Association of patient’s data with SNPs

Analysis between the genotypes of *PADI4* gene rs11203367 and rs1748033 SNPs and data of RA patients, including PADI4 mRNA expression, age, BMI, anti-CCP, RF, ESR, CRP, and DAS28 was conducted (Table 5). The patient’s data were not significantly different among RA patients with three genotypes for rs11203367 SNP. Anti-CCP level was higher significantly in TT carriers for rs1748033 polymorphism (*P* = 0.041). Furthermore, in RA patients with TT genotype for rs1748033 SNP, there was a statistically significant higher DAS28 score (*P* = 0.034).

**Table 5:** Association of *PADI4* gene rs11203367 and rs1748033 SNPs with clinical manifestations of RA patients

Characteristic	rs11203367 (TT)	rs11203367 (TC)	rs11203367 (CC)	*P* value
PADI4 mRNA expression	2.65 ± 1.33	2.78 ± 1.16	2.60 ± 1.32	0.416
Age	50.31 ± 11.24	55.23 ± 10.26	53.56 ± 11.56	0.216
BMI	30.2 ± 5.12	28.41 ± 4.12	29.25 ± 4.89	0.455
Anti-CCP	56.31 ± 41.49	57.56 ± 42.54	58.21 ± 41.41	0.208
RF	41.33 ± 31.64	44.34 ± 33.25	39.23 ± 31.45	0.190
ESR	20.15 ± 18.21	22.3 ± 18.45	21.68 ± 18.90	0.244
CRP	3.41 ± 2.45	3.58 ± 2.55	2.41 ± 2.61	0.416
DAS28	4.89 ± 1.01	3.89 ± 1.16	4.7 ± 1.18	0.129
	**rs1748033 (TT)**	**rs1748033 (TC)**	**rs1748033 (CC)**	
PADI4 mRNA expression	2.81 ± 1.18	2.61 ± 1.27	2.66 ± 1.38	0.311
Age	51.48 ± 10.24	52.89 ± 10.47	53.74 ± 10.25	0.657
BMI	28.95 ± 5.01	29.51 ± 5.18	29.84 ± 5.11	0.587
Anti-CCP	64.54 ± 45.25	55.78 ± 40.44	54.73 ± 38.25	**0.041**
RF	44.07 ± 34.15	41.28 ± 30.81	40.18 ± 30.07	0.077
ESR	21.14 ± 18.78	21.45 ± 17.91	21.20 ± 18.12	0.619
CRP	3.14 ± 2.23	3.61 ± 2.24	2.51 ± 2.67	0.358
DAS28	6.95 ± 1.74	4.41 ± 0.88	3.10 ± 0.46	**0.034**

PADI4; Peptidyl Arginine Deiminase 4, BMI; Body mass index, DAS28; Disease Activity Score 28, RF; Rheumatoid factor, Anti-CCP; Anti-Cyclic Citrullinated Peptide Antibody, ESR; Erythrocyte sedimentation rate, CRP; C-reactive protein

The analysis was also repeated based on gender stratification and smoking status, yielding no statistically significant findings (data not shown).

## Discussion

A bulk of investigations have revealed that genetic abnormalities are involved in RA etiopathogenesis and account for approximately 60% of inheritance risk of RA as well as clinical presentations of RA disease [28–31]. Among the non-HLA genes associated with RA susceptibility, polymorphisms in the *PADI4* gene have been repetitively investigated in association with RA risk in different ethnicities in the world. In addition, a multi-ancestry large-scale genome-wide association study (GWAS) of 276,020 samples published in 2022 revealed involvement of 34 novel loci in association with RA susceptibility, suggesting essential roles of the immune system and joint tissues in etiology and pathogenesis of RA [32].

The *PADI4* gene is located on the short arm of chromosome 1 that encodes the enzyme PADI4. In the presence of calcium, this enzyme catalyzes the citrullination of histone proteins at specific sites of the histone H3 and H4 tails by converting arginine to citrulline, which alters the structure and functional properties of proteins. Citrulline proteins are detected by citrulline cyclic anti-peptide serum antibodies, which are the most specific autoantibodies in patients with RA [33]. Following the function of the PADI4 enzyme, protein citrulline and the production of anti-citrulline antibodies occur in the joint. These events indicate a very close relationship between protein citrullination and altered antigenicity of peptides and autoimmune pathogenicity in RA [34].

Over the course of past years, both GWASs as well as sporadic association studies worldwide have disclosed the involvement of PADI4 genetic polymorphisms in altering the risk of RA susceptibility. Based on the initial investigations by Suzuki et al., assessment of the SNPs distributed across the *PADI4* gene in Japanese patients disclosed that the rs2240340 polymorphism was the most important SNP associated with RA susceptibility [13]. Nonetheless, numerous studies implemented on the Caucasian, European, as well as East Asian populations to validate this finding resulted in inconsistent outcomes. In order to resolve the inconsistencies regarding the association of *PADI4* gene polymorphisms with RA, a meta-analysis study was performed by Lee et al. in 2007. This study included both Asian and European studies to evaluate the potential association of the *PADI4* gene polymorphisms with RA proneness [21]. According to this meta-analysis, it was detected that there were significant associations of PADI4-104 (rs1748033), PADI4-94 (rs2240340), and PADI4-90 (rs11203367) polymorphisms with RA risk in Asian population. However, only PADI4-94 (rs2240340) was significantly associated with RA susceptibility in European population. This meta-analysis indicated that PADI4 SNPs might be more important contributors for RA risk in Asian population. It was contemplated that there was no strong power in the European studies due to small sample sizes and hence were unable to identify PADI4 genetic associations with RA disease. Therefore, a large-scale study in the UK population (19,000 UK patients) was performed, but no significant association was identified between PADI4-94 SNP and RA was found [35]. Hou et al. performed a meta-analysis on RA patients with Asian and European ethnicities and identified that PADI4-89, PADI4-90, PADI4-92, PADI4-94, PADI4-100, PADI4-104 polymorphisms were significantly associated with RA risk in the Asian population [22]. Additionally, the 2015 meta-analysis by Lee and colleagues indicated association of *PADI4* gene rs11203367 SNP with increased RA risk in the overall as well as Asian populations [36]. Replication study in a Southern Mexican population indicated that the T allele of rs11203367 was associated with increased (OR = 1.35) RA proneness [37].

A study in Iranian population involving 665 RA patients and 392 sex-, age-, and ethnicity-matched healthy individuals did not indicate association of *PADI4* gene rs11203367 and rs874881 polymorphisms with RA risk [23]. Our investigation also revealed that there was no significant association of the *PADI4* gene rs11203367 SNP with RA risk in our population. Furthermore, no association was identified between this SNP and clinical manifestations as well as demographics of the patients. Our research also identified that *PADI4* gene rs11203367 did not impress the mRNA expression of PADI-4 in the serum samples from RA patients.

A study on the RA population from Southeast Iran revealed that *PADI4* rs1748033 T allele as well as the dominant and codominant inheritance models were significantly associated with increased RA risk [24]. Our investigation also indicated that the mutant allele, TT and TC genotypes, and dominant (TT + TC vs. CC) and recessive (TT vs. TC + CC) models were associated with higher risk of RA. Interestingly, anti-CCP and DAS28 was higher significantly in TT carriers for rs1748033 polymorphism. Even though the mRNA expression of PADI4 in the serum samples was not associated with genotypes of rs1748033 SNP, it might be involved in determining the clinical picture of RA. It is suggested to investigate the association of rs1748033 SNP with PADI4 in better clinical samples as well as in protein levels. It should be noted that mRNA expression of PADI-4 in the serum samples from RA cases was significantly correlated with Anti-CCP, RF, CRP, and DAS28. Hence, even though PADI-4 might be involved in the pathogenesis of RA, it is seemingly not impressed by genetic polymorphisms rs11203367 and rs1748033 in the *PADI4* gene.

Previous reports show that PADI4 polymorphisms might interact with smoking status as an environmental factor in RA cases, especially male subjects [38]. We also performed the analysis based on smoking and gender status. However, no significant results were yielded. Furthermore, the mRNA expression of PADI4 was not significantly higher when smoker patients were compared. This implies to the genetic composition of different populations, and here in our population smoking was not a contributing environmental factor in accompany with PADI4 SNPs in promoting the proneness of individuals to develop RA disease.

This study had some limitations and caveats; first, we did not evaluate other SNPs of *PADI4* gene in association with RA disease as different SNPs might collectively in form of haplotypes might impress disease risk. Second, individuals of this study were recruited from one center in our hospital, therefore it would be far interesting to investigate the *PADI4* gene SNPs in a large national population to resolve potential bias. Third, we did not control the mRNA expression of PADI4 for confounding factors like therapeutic regimen of the RA patients.

In summary, *PADI4* gene rs1748033 SNP had association with increased RA proneness in an Iranian population. In addition, this polymorphism might affect the RA pathogenesis regardless of impressing the levels of PADI-4 in the serum of RA patients. Further investigations with large sample size obtained across the whole country might be contributing in identification of the PADI4 genetic polymorphisms with RA risk in the Iranian population.

## Data Availability

The authors are unable to share detailed clinical data due to full anonymization of the data is very difficult. But the datasets analyzed and generated during the study are available from the corresponding author on reasonable request.

## Abbreviation

RA: Rheumatoid arthritis
HLA: Human leukocyte antigen
SNP: Single nucleotide polymorphisms
PADI4: Peptidyl arginine deiminase 4
CCP: Cyclic citrullinated peptide
ACR: American College of Rheumatology
EULAR: European Alliance of Associations for Rheumatology
DAS28: Disease Activity Score 28
GAPDH: Glyceraldehyde 3-phosphate dehydrogenase
OR: Odds ratio
CI: Confidence interval
HWE: Hardy–Weinberg Equilibrium
SPSS: Statistical Program for Social Science
SD: Standard deviation
RF: Rheumatoid factor
ESR: Erythrocyte sedimentation rate
CRP: C-reactive protein
BMI: Body mass index
GWAS: Genome-wide association studies
